# Radiotherapy after surgery for spinal metastasis is associated with superior neurological improvement as compared to surgery alone

**DOI:** 10.1051/sicotj/2025026

**Published:** 2025-05-12

**Authors:** Michael G. Kontakis, Jessica Ehne, Sayam Svahn-Karahan, Panagiotis Tsagkozis

**Affiliations:** 1 Department of Surgical Sciences, Orthopaedics, Uppsala University Akademiska sjukhuset, ingång 61 6 tr 751 85 Uppsala Sweden; 2 Department of Molecular Medicine and Surgery, Karolinska Institute K1 Molekylär medicin och kirurgi, K1 MMK Ortopedi 171 76 Stockholm Sweden; 3 Department of Acute and Reparative Medicine, Karolinska University Hospital K1 Molekylär medicin och kirurgi, K1 MMK Ortopedi 171 76 Stockholm Sweden

**Keywords:** Spine, Metastasis, Surgery, Radiotherapy, Recovery

## Abstract

*Introduction:* Treatment of spinal metastases is multidisciplinary, where radiotherapy (RT) and surgery have a central role. The effect of adjuvant post-operative RT versus surgery alone for metastatic spinal disease has not been previously investigated. Our aim was to analyze whether post-operative RT was associated with better functional outcome or increased incidence of local complications after surgical treatment for spinal metastatic disease. *Methods:* Information on neurologic outcome of 200 patients surgically treated for spinal metastases was retrieved from the institutional registry. The events of pre-operative and post-operative neurological function, post-operative wound complications as well as death and implant revision were available. *Results:* Post-operative RT was significantly associated to superior neurological recovery, evaluated both as restoration of the ambulatory capacity and absolute change in the Frankel score. At the same time, use of post-operative RT was not associated to an increased risk of wound complications. The risk for revision surgery when RT was administered was similar to surgery alone in a competing risks analysis with death as the competing event. *Discussion:* The results indicate that surgery with post-operative RT is associated with superior neurologic recovery than surgery alone. The results also do not indicate any significant risk for wound healing problems with administered post-operative RT.

## Introduction

The vertebral column is a common location for metastases, as autopsy reports have suggested the presence of spinal metastases in up to 70% of patients with metastatic disease [1]. Previously, spinal metastases were often treated with radiotherapy (RT) only, but treatment has shifted towards the more liberal use of surgery since data suggest superior result regarding the restoration of ambulatory capacity when combining surgery and RT [2, 3]. Although the use of RT after surgery for metastatic bone disease is poorly substantiated [4], in the case of spinal metastases, RT is routinely recommended after decompressive surgery. Studies have shown good local control when RT is administered early after surgery, while other studies have correlated RT with post-operative survival. However, there are no studies directly comparing the outcome of surgery followed by RT as compared to surgery alone regarding the primary functional outcome, which is the neurological recovery.

Although RT inhibits local tumour progression [5], which is highly desirable in the case of spinal metastases since surgery entails as a rule only debulking of the tumour and not complete resection [6, 7], it may theoretically cause wound complications and surgical site infection [8, 9]. Surgical site infections have previously been found to comprise the most common complication in spinal metastatic surgery, occurring in up to 10% of spine surgeries [10].

In the present retrospective study, we aimed to investigate whether RT after surgery for metastatic disease of the spine is associated with better neurologic outcome as compared to surgery alone. Furthermore, we analysed the association between post-operative RT and the development of local wound problems and infection, as well as the need to undergo revision surgery.

## Material and methods

### Study design and study population

This is a retrospective registry-based cohort study using patient data for metastatic bone disease of the spine. Information was retrieved from the institutional database to identify patients surgically treated for spinal metastases.

The study cohort comprised 200 patients which have been surgically treated for between years 2000 and 2021. The demographic characteristics of the study population and the relevant treatment data are presented in Table 1. Of the 200 patients, 128 received RT post-operatively. Surgery without subsequent RT was performed on 72 patients. Median overall patient survival was 8 (3–23) months. Patients who received post-operative RT had higher performance scores as indicated by the mean value of the modified Tokuhashi score (Table 2), indicating a treatment bias for RT in more medically fit patients.

**Table 1:** Demographic and clinical parameters of the patients.

Demographic and clinical parameters	
Males/Females	128/72
Age (mean ± SD)	65 ± 13
Post-operative radiotherapy (Yes/No)	128/72
Instrumentation	
Total posterior fusions/anterior fusions	83/7
No fusions	101
Extent of disease	
Single skeletal	35
Numerous skeletal	61
Generalized	93

**Table 2:** Gender, age, and Tokuhashi score of the patients that did not receive, versus those that received radiotherapy. ^a^Pearson’s *χ*^2^ test.

	Post-operative radiotherapy		
	No	Yes	*p*-value
Males	46	82	
Females	26	46	1^a^
Age (mean ± SD)	66 ± 12	64 ± 12	0.34
Tokuhashi (mean ± SD)	1.14 ± 0.3	1.33 ± 0.47	0.004

### Follow-up and clinical outcome

Patient neurology was classified according to the Frankel scale, both pre-operatively and post-operatively. Information on the neurological outcome at the late follow-up was available at a time-point of 6–9 months after surgery. The modified Tokuhashi score (ranging from 1 to 12) was divided into two categories based on surgical recommendations. Scores below 9 were assigned a value of 1, while scores of 9 or higher were assigned a value of 2. Patients with a Frankel score of D–E were designated as ambulatory, and these with Frankel score of A–C as non-ambulatory. Complications were only categorized and not further described in the registry and were therefore only descriptively reported revision surgery was defined as any surgical procedure which involved change or removal of an implant used, or the use of an implant in cases of previous surgery without any instrumentation.

### Statistical analysis

The data are presented as medians (25th–75th quantile ranges) or as means ± SDs. Overall patient survival and revision surgery rate were calculated using the Kaplan-Meier method and the log-rank test was used for comparisons between groups. For survival analysis, the Cox Proportional Hazards Model was performed. Neurological outcomes in Frankel scale were converted to ordinal numerical values and comparison between groups was done using the Mann-Whitney *U*-test. Ambulatory capacity was divided in two groups (independently ambulating or not, as designated previously) and comparisons between groups were done using the χ^2^ test. The competing risks regression function was also used to assess whether RT was associated with a reduced risk of implant revision. Statistical tests (with a level of significance of *p* < 0.05) were performed and graphs were generated with R (packages: ggplot2, survival, survminer, cmprsk) [11].

## Results

### Functional outcome

Post-operative RT as compared to surgery only resulted in superior neurologic recovery. One hundred thirty-one patients were non-ambulatory prior to surgery. As shown in Table 3, 72 of them recovered their ambulatory capacity, most (*n* = 58) having received post-operative RT (*p* < 0.001). Of the 69 patients that were ambulatory prior to surgery, 60 retained this function, which was not associated to the use of post-operative RT (*p* = 0.874). There was no association between the use of post-operative RT and the ambulatory status of patients prior to surgery (*p* = 0.62).

**Table 3:** Ambulatory status and post-operative radiotherapy.

Preoperative status	Radiotherapy postoperatively	Ambulatory postoperatively	Non-ambulatory postoperatively	*P*-value
Ambulatory postoperatively	Yes	35	5	
	No	25	4	0.874
Non-ambulatory postoperatively	Yes	58	30	
	No	14	29	<0.001

**Figure 1:**
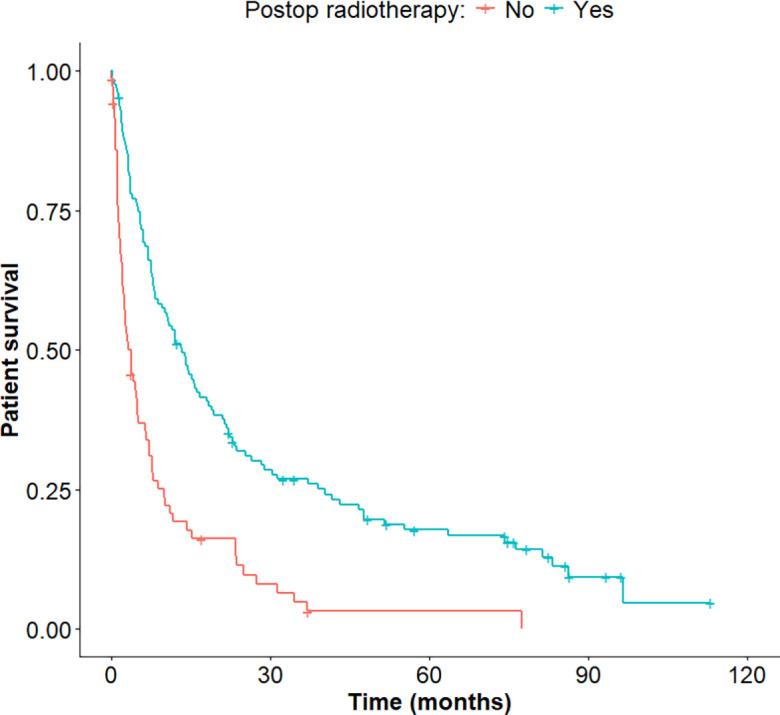
Dot-plot of the neurologic outcome after surgery for metastatic spinal disease, with or without post-operative radiotherapy.

**Figure 2:**
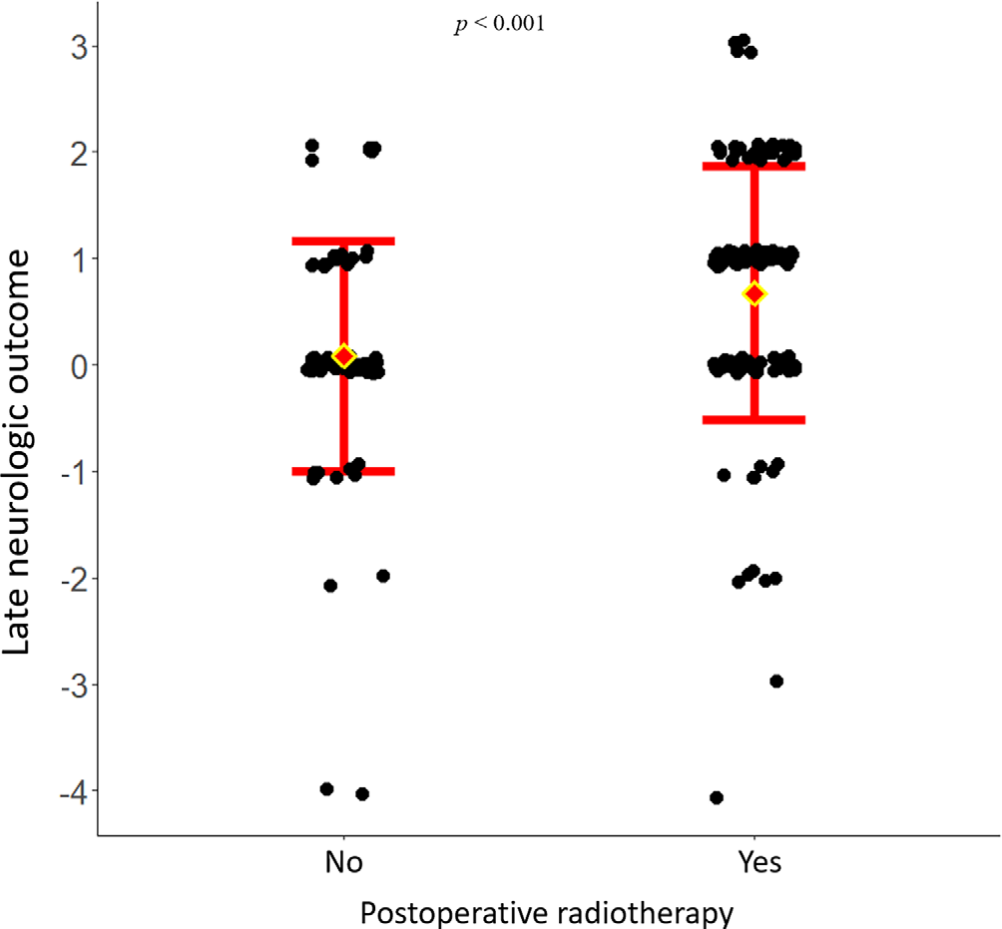
Survival plot after Kaplan-Meier analysis of overall survival of 200 patients surgically treated for metastatic disease of the spine, depending on the administration of post-operative radiotherapy or not.

When comparing the absolute change in the neurological function as graded in the Frankel scale, and as illustrated in Figure 1, use of post-operative RT was associated with superior improvement (*p* < 0.001). Patients who received post-operative RT had a better oncologic outcome than the ones who did not (*p* < 0.001), with a hazard ratio of 0.41 [0.29–0.56] (Figure 2).

### Complications and revision surgery

**Figure 3:**
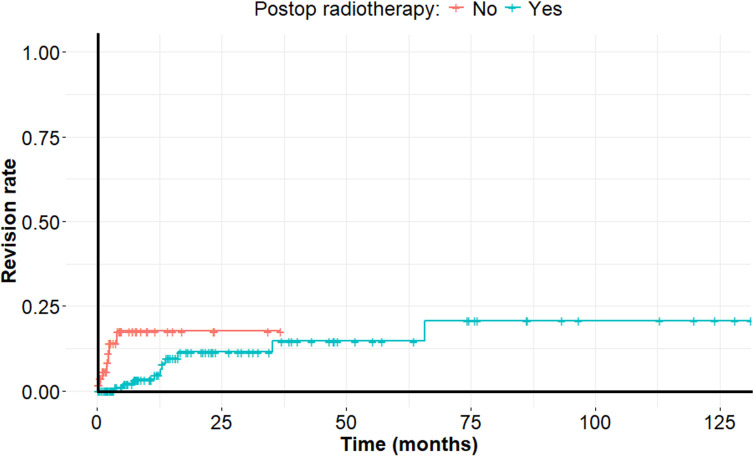
Rate of revision surgery for metastatic disease of the spine, depending on the use of post-operative radiotherapy.

Post-operative RT did not result in increased wound healing problems and did not lower the risk for revision surgery. Fifteen out of 200 patients (7%) experienced wound complications (dehiscence and infection), and there was no association between the administration of RT and the incidence of wound complications (*p* = 0.24). Revision surgery was reported in 17 cases in total. In a competing risks model (Figure 3), with death as the competing risks factor, the use of post-operative RT was associated with a 38% reduction in the risk of implant revision rate, that however did not reach statistical significance (*p* = 0.33).

## Discussion

Whether post-operative RT improves the functional outcome of decompressive surgery for spinal metastasis with neurological deficits is unknown. In our retrospective registry study, we showed that RT given after surgery was superior to surgery alone regarding neurological recovery, without an increased risk for wound healing problems. To our knowledge, this is one of the first studies to directly examine this association, albeit retrospectively. We believe that the study benefited from comprising a comparatively large cohort and acknowledge its retrospective design with inherent treatment bias and confounding factors as major limitations. Selection bias is obviously present in this association since the analysis is retrospective and to our opinion constitutes the main shortcoming of this study. Patients of already poor health status and/or prognosis were less likely subjected to post-operative radiotherapy. Such a selection bias was obvious when comparing the overall survival between these two subgroups, where the group of patients who received RT had longer post-operative survival. Individuals with poor expected survival might therefore have an *a priori* worse chance of recovering their ambulatory function, due to less intense rehabilitation, and simply less available time to recover from their deficit.

Despite these limitations, we documented a clear association of post-operative RT with a better neurological outcome in terms of restoration of the ambulatory capacity for patients that were non ambulatory prior to surgery. As complete tumour excision is almost never achieved during classical decompressive surgery for spinal metastases [6, 7], it is reasonable to suggest that subsequent RT helps control the growth of the tumour residue left after the surgical resection [5]. The fact that this effect was obvious in patients who had more severe neurological compromise prior to surgery probably implies that surgery alone is adequate in minor compression of the cord. Notably, studies regarding the effect of the intensity and quality of rehabilitation on the functional outcome of patients with metastatic spinal disease are lacking. Another aspect of post-operative RT, that we were not able to assess in our study, is its effect on pain reduction. RT may have direct analgesic effects and decrease local pain due to inhibition of tumour progression [12].

The secondary finding of this study is that post-operative RT did not increase the risk for wound healing problems and infections. In line with previous research on spinal metastasis surgery, surgical site infection constituted the most common complication in our sample. This corroborates findings of previous research which has reported similar incidence following spinal metastasis surgery [10, 13]. However, this risk does not seem to be dependent on the use of post-operative RT, probably because the radiation doses used are relatively low [2].

RT was associated with a tendency to lower revision surgery rate, an association which however did not reach statistical significance in a competing risks survival model. A larger cohort is obviously needed to investigate this hypothesis. If true, this effect may be explained by the lower failure rate due to decreased local progression of the tumour [14], and would provide even stronger evidence for using post-operative RT.

Overall, given the absence of prospective randomized trials our data support the use of routine post-operative RT after surgery for spinal metastasis, and we advocate that it should be offered to all patients who are medically capable of such a treatment, since it confers a benefit regarding neurological recovery and possibly reduces the risk for revision surgery in patients with good prognosis, whereas at the same time does not entail significant risks for wound complications.

## Conclusion

This study provides evidence supporting the use of routine post-operative RT following surgery for spinal metastases. RT may be associated with improved neurological outcomes, particularly in restoring ambulatory function. Complication rates, particularly surgical site infections, did not appear to be significantly influenced by post-operative RT, likely due to the relatively low radiation doses used. Post-operative RT was linked to a tendency toward lower revision surgery rates, though this did not reach statistical significance. If larger studies confirm this finding, it would further justify the routine use of RT by reducing tumour progression and the need for additional surgical interventions. Given the lack of prospective randomized trials, our findings advocate for the routine use of post-operative RT in medically eligible patients. It offers clear benefits in terms of neurological recovery and potentially reduces the risk of revision surgery, without increasing wound-related complications.

## Data Availability

The data are available upon reasonable request by the corresponding author.
